# Predictive Factors of Response to Biological Disease Modifying Antirheumatic Drugs: Towards Personalized Medicine 

**DOI:** 10.1155/2014/386148

**Published:** 2014-01-12

**Authors:** Claire I. Daïen, Jacques Morel

**Affiliations:** Department of Rheumatology, Lapeyronie Hospital, University Montpellier I-II and IGMM-UMR5535, CNRS 1919, Route de Mende, 34295 Montpellier Cedex 5, France

## Abstract

Many therapies are now available for patients with rheumatoid arthritis (RA) who have an inadequate response to methotrexate including tumor necrosis factor inhibitors, abatacept, tocilizumab, and rituximab. Clinical response to drugs varies widely between individuals. A part of this variability is due to the characteristics of the patient such as age, gender, concomitant therapies, body mass index, or smoking status. Clinical response also depends on disease characteristics including disease activity and severity and presence of autoantibodies. Genetic background, cytokine levels, and immune cell phenotypes could also influence biological therapy response. This review summarizes the impact of all those parameters on response to biological therapies.

## 1. Introduction

Many biological therapies are now available for patients with rheumatoid arthritis (RA) who have an inadequate response to synthetic disease modifying antirheumatic drugs (sDMARD) especially methotrexate (MTX) or to a first tumor necrosis factor (TNF) inhibitor (TNFi). They can be treated with either TNFi (etanercept (ETN), infliximab (IFX), adalimumab (ADA), certolizumab, or golimumab), or a T cell targeting therapy (CTLA4-Ig abatacept (ABA)) or an anti-IL-6 receptor drug (tocilizumab (TCZ)), or a B cell targeting therapy, mostly represented by anti-CD20 antibodies like rituximab (RTX). Clinical response to drugs varies widely between individuals. A part of this variability is due to drug concentration and pharmacokinetic which is influenced by the characteristics of the patient such as age, gender, renal and liver functions, body mass index (BMI), or smoking status. Concomitant therapies and drug immunogenicity also influence drug concentrations. Clinical response depends on disease state and disease characteristics as well. Indeed, there are different subtypes of RA with different genetic backgrounds, that is, seropositive or seronegative RA [1] and benign or destructive RA [2–4]. Depending on patients, the RA could be preferentially mediated by one cytokine; for example, some diseases are very dependent on TNF, whereas others are not [5]. One immune cell type can also be more important in some patients than others (i.e., B or T cells, Th1 or Th17 [6], etc.). Although all these parameters may influence therapeutic response, tools which could be used in daily practice to predict response to biological drugs are lacking. This review synthesizes the largest studies on factors influencing response to TNFi, ABA, RTX, and TCZ therapy (Table 1).

## 2. Place of Patient's Characteristics in Predicting Response to Biological Therapies

Several studies have assessed the value of age, gender, concomitant drugs, body mass index (BMI), or smoking status for predicting biological DMARD response. Most of them concerned TNFi.

### 2.1. Age, Gender, and Concomitant Drugs

#### 2.1.1. TNFi

Several studies showed that male patients are more likely to respond to TNFi or to achieve remission with TNFi. Those studies include Kleinert's work that evaluated the effectiveness of ADA in 2,625 RA patients (*β*2 weight = −0.182, partial *r* ^2^ = 0.003; *P* = 0.003) [7], the Research in Active RA trial (ReAct), a 12-week study open label on ADA that enrolled 6,610 RA patients (HR = 1.284; 95% CI = 1.160–1.422; *P* = 0.0001) [8], and the Trial of Etanercept and Methotrexate with Radiographic Patient Outcomes (TEMPO) that included 682 patients receiving ETN (OR = 1.92; 95% CI = 1.32–2.77) [9]. Younger patients were found to have better clinical outcomes in Kleinert's study (*β*2 weight = 0.012, partial *r* ^2^ = 0.014; *P* < 0.001) [7] and in ReAct (>75 years versus <40 years: HR = 0.611; 95% CI = 0.461–0.810, *P* = 0.0006) [8]. Conversely, no association with gender or age and clinical response was found in the British Society for Rheumatology Biologics Register (BSRBR) [10] and in the retrospective South Swedish Arthritis Treatment Group Register GISEA [11]. The use of MTX was associated with good clinical outcomes in many different studies including BSRBR [10], Kleinert's study [7], GISEA [11], and ReAct [8].

#### 2.1.2. Other Biological Therapies

Concerning TCZ, the Japanese multicenter retrospective study (REACTION) involving 229 patients revealed that younger age was independently associated with a good EULAR response and remission at 24 weeks [12]. No other factors appeared to have a statistically significant predictive value for remission. In 104 RA patient included in DANBIO registry and treated with ABA, higher age was associated with EULAR good-or-moderate response (OR = 1.04/year increase (95% CI 1.01 to 1.08/year), *P* = 0.012) [13]. Conversely, in the Orencia and Rheumatoid Arthritis (ORA), prospective registry which included 558 patients with RA, age, gender, and concomitant sDMARD did not significantly differentiate between EULAR responders and nonresponders [14]. In the 540 RTX-treated patients included in BSRBR who had experimented at least one TNFi failure, female sex was significantly associated with lower odds of disease remission (0.45 (95% CI 0.12, 0.78)) [15].

### 2.2. Body Mass Index

The influence of BMI on therapeutic response at 16 weeks was evaluated in 89 RA patients treated with IFX 3 mg/kg [16]. BMI correlated positively with DAS28 at baseline. A negative association between BMI and the absolute decrease of DAS28 was found (*P* = 0.001). In GISEA, DAS28-remission at 12 months was noted in 15.2% of the obese subjects, in 30.4% of the patients with a BMI of 25–30 kg/m^2^, and in 32.9% of the patients with a BMI of <25 kg/m^2^ (*P* = 0.01) [17]. The difference in terms of remission percentage between obese patients and others was significant only in IFX-treated patients (not in ADA- and ETN-treated patients).

### 2.3. Smoking Status

There was a significant association between current cigarette smoking and a lower response in patients receiving IFX (OR 0.77 (95% CI 0.60–0.99)) in the BSRBR [18]. This result was confirmed in a retrospective case control study of 395 RA [19], in a prospective cohort of 617 Portuguese [20] and in a Swedish register that included 1,998 early RA (Epidemiological Investigation of Rheumatoid Arthritis EIRA) [21]. However, it has not been demonstrated that smoking cessation increases the chance of response to therapies. In a Swedish study on 1,460 RA patients with disease duration ≤2 years (BARFOT (better anti-rheumatic pharmacotherapy)), 127 patients quitted smoking after inclusion in the study. Smoking cessation was negatively associated with EULAR response at 8 years (OR 0.44 (0.22–0.86); *P* = 0.02). To our knowledge, no data are currently available on the influence of smoking on response to TCZ, ABA, or RTX.

In summary, male gender, younger age, and nonsmoking status are better predictors of response or remission for patients starting a TNFi (Table 2). Concomitant use of MTX improves drug response. Obese patients are less likely to achieve remission with IFX. Only few data are available for TCZ, ABA, or RTX. The gender can influence the disease phenotype in RA. Male patients who have a later onset of RA are more likely to be seropositive for RF and have higher titers of anticitrullinated peptide antibodies compared with female patients [22, 23]. They are also more likely to have a history of smoking and to carry the HLA-DRB1 shared epitope. Moreover, when comparing patients with a similar degree of radiographic joint destruction, women have worse scores for DAS28 and HAQ than men, possibly due to higher pain perception or an overestimate by men of their functional capacity [24]. Smoking is associated with a more severe disease and therefore probably more resistant to therapy [25, 26]. It also directly influences drug metabolism by inducing cytochrome P450 activity which was shown to impact other drug responses [27]. The concomitant use of MTX largely improves response through synergic actions of the drugs on RA but also probably by its impact on drug immunogenicity since the occurrence of antidrug antibodies is less frequent with MTX combined with biological therapies [28]. BMI influences drug concentration but is also associated with RA disease activity. Indeed DAS28 score increases with BMI in women [16, 22].

## 3. Place of RA Characteristics in Predicting Response to Biological Therapies

RA characteristics such as disease duration, disease activity score (DAS28), functional index (Health Assessment Quality (HAQ)), and previous therapeutics can influence drug response or presence of autoantibodies (rheumatoid factor (RF) and anticitrullinated peptide antibodies (ACPA)).

### 3.1. DAS28, HAQ, and Previous Therapeutics

#### 3.1.1. TNFi

In most studies with TNFi, patients with higher baseline HAQ scores are less likely to respond or to achieve remission [8–10, 20]. In the British registry BSRBR, the Odd Ratio for EULAR response was 0.59 (95% CI 0.50–0.69) per unit increase in HAQ. Moreover, high DAS28 at baseline is a good predictor of DAS28 decrease [7, 11] but is inversely associated with EULAR remission [8, 9, 11]. Multiple previous biologics are associated with a reduced therapeutic response [7, 8].

#### 3.1.2. Other Biological Therapies

Similar finding were found for other therapies. In the 97 patients treated with TCZ registered in the nationwide Danish DANBIO registry, lower HAQ score at baseline was associated with EULAR response (OR = 2.51 (1.04–6.04), *P* = 0.041) [13] and higher DAS28 at baseline was significantly associated with achieving a low DAS28 (OR = 0.48 (0.32–0.73), *P* < 0.001) [13]. In ORA registry, initial DAS28 was higher in ABA responders (5.4 (4.7–6.5)) than in nonresponders (4.9 (4.0–6.0), *P* < 0.0001) [14]. For RTX, several studies also showed association between EULAR response and low HAQ, high DAS28 and low number of previous biological agents [15, 29, 30].

### 3.2. Autoantibodies

#### 3.2.1. TNFi

Rheumatoid factor (RF) status was neither significantly associated with response to TNFi in BSRBR [10] nor with remission in ReAct and TEMPO studies [8, 9]. Presence of RF or ACPA was found to predict a reduced likelihood of treatment response [20, 31]. Rheumatoid factor negative patients had a greater mean improvement in DAS28 (+0.48; 0.08–0.87) compared to RF positive patients at 6 months after adjusting for DAS28, HAQ, current DMARD therapy, and gender in BSRBR.

#### 3.2.2. Other Biological Therapies

A meta-analysis of 23 clinical trials and observational studies showed that RF positivity at baseline predicts better ACR20 (OR, 1.95 (1.24, 3.08)), ACR50 (OR, 5.38 (2.50, 11.60)), and EULAR response (OR, 3.52 (1.66, 7.45)) in 14 studies with RTX and better ACR20 (OR, 1.51 (1.21, 1.90)) in 6 studies with TCZ [32]. In 3 studies with ABA, no association was found between response and RF (OR 1.36 (0.97, 1.90)).

In summary, higher DAS28 values at baseline are associated with response to biological therapies, which can be explained by a better chance of decreasing DAS28 of at least 0.6 if initial values are high. Conversely, patients with higher DAS28 values at baseline are less likely to achieve remission. High HAQ values and high numbers of previous biological therapies are associated with decreased chance of response which can be explained by more aggressive diseases. The presence of RF ± ACPA increases the chance of responding to RTX and to a lesser extent to ABA. Conversely, RF ± ACPA positive status negatively influences the chance of TNFi response. Autoantibodies status could have impact on clinician choice. However strategy based on autoantibody positivity to introduce RTX rather than another biological drug still needs to be validated.

## 4. Place of Drug Concentrations in Predicting Response to Biological Therapies

Plasmatic concentrations of TNFi are known to influence therapeutic response. This was shown in patients treated with IFX [33], ADA [34], and ETN [35]. Previous studies found that the presence of neutralizing antidrug antibodies is associated with a reduction of drug levels below the therapeutic range and a suboptimal clinical outcome. Those antidrug antibodies are found in about 30% of the patients treated with monoclonal antibodies (IFX and ADA) and in a lower proportion (0–5%) in patients treated with the soluble receptors (ETN) [35–37]. Drug concentration and antidrug antibody monitoring could also be used as a predictor of response to TNFi. We previously showed in 18 RA patients that 3-month ETN levels correlated significantly with change in DAS28 between baseline and 6 months (*r* = −0.62, *P* = 0.006) [38]. Thus, measures of ETN concentration could help in the decision to continue or not the treatment in patients who have an insufficient response to treatment after 3 months. In a cohort of 292 RA patients treated with ETN, Jamnitski et al. compared the response of patients for whom ETN was the 1st TNFi and others. Eighty-nine out of 292 of these patients were switchers and had previously been treated with either IFX (*N* = 30) or adalimumab (*N* = 59). Among the switchers, 53% of them had antidrug antibodies at baseline. TNFi naive patients responded better to ETN compared to switchers without antidrug antibodies after 16 weeks of etanercept treatment (*n* = 42) [39]. Conversely, DAS28 improvement in switchers with antidrug antibodies (*n* = 47) did not differ from the one of TNFi naive patients. Thus in patients whose inadequate response to a first TNFi is not explained by antidrug antibodies, the disease seems to be mediated by other mechanisms than TNF and switching to another class of biologics, such as TCZ, ABA, or RTX, would be a better option.

## 5. Place of Genetics in Predicting Response to Biological Therapies

Many different single nucleotide polymorphisms (SNPs) were suggested to be associated with response to biological therapies, but very few have been confirmed. We voluntarily present here only data that have been replicated at least in another cohort.

### 5.1. TNFi

Using candidate gene approach, SNPs related to TNF*α* or TNF*α* receptors (TNFR) were studied. In 2003, it was found that in 59 RA patients with TNFA-308G/G (rs1800629) responded better to IFX than those with A/A or A/G genotypes (12847678) (OR = 1.93; *P* = 0.009) [40]. Some other studies confirmed this data whereas others did not. A meta-analysis that included 11 studies and 2579 patients did not find a significant association between TNFA-308 and TNFi response. Thirty-one SNPs associated with the risk of RA (i.e., TNFAIP3, STAT4, PTPN22, HLA class II, etc.) were analyzed in 1,283 RA patients [41]. The SNP at the CD45 (also called PTPRC) gene locus (rs10919563) was associated with EULAR good response versus no response (OR = 0.55, *P* = 0.0001) in multivariate analysis. This was confirmed in another large independent cohort of 1,115 English patients (OR = 0.62 (0.40–0.95), *P* = 0.03) [42].

In genome-wide association studies (GWAS) on 566 anti-TNF-treated RA patients, association with treatment response was found for 171 genotyped markers [43]. Seven of them were corroborate in the combined analysis of 2 independent replication cohorts (*n* = 379 and *n* = 341). The strongest effect was at rs17301249, mapping to the EYA4 gene on chromosome 6: the minor allele conferred improved response to treatment (coefficient −0.27, *P* = 5.67 · 10^−5^). The minor allele of rs1532269, mapping to the PDZD2 gene, was associated with a reduced treatment response (coefficient 0.20, *P* = 7.37 · 10^−4^). The remaining associated SNPs mapped to intergenic regions on chromosomes 1 (rs12081765), 4 (rs4694890), 11 (rs1350948), and 12 (rs7305646 and rs7962316) [43].

### 5.2. Other Biological Therapies

158V/F SNP of FCGR3A was shown to be associated with response to RTX in 111 patients included in an ancillary study of SMART [44]. V allele carriage independently associated with response to RTX (OR 3.8 (1.2–11.7), *P* = 0.023) in multivariate analysis. This was recently confirmed in a multicenter Italian study including 212 RA patients, with 89.5% of response at 6 months for VV versus 66% for VF and in 66.2% for FF patients (*P* = 0.01) [45]. Probability of response at 6 months was very high when at least two of the three following items were present: positive rheumatoid factor and/or anticyclic citrullinated peptide antibodies, previous treatment with ≤1 TNFi, and 158VV FCGR3A genotype (*P* < 0.0001; OR 7.9, 95% CI 4.1 to 15.1). Similar results were found in non-Hodgkin lymphomas [46]. However, in an American study on 158 RA patients, FCGR3A 158V/F SNP was not associated with response to RTX [47].

In summary, CD45 (rs10919563), EYA4 (rs17301249), and PDZD2 (rs1532269) SNPs are associated in independent cohorts to TNFi response. The role of these different SNPs remained to be further studied. 158VV FCGR3A is associated with RTX response in European countries.

## 6. Place of Cytokines and Immune Cell Assessment in Predicting Response to Biodrugs 

### 6.1. TNFi

One could speculate that a higher level of TNF-*α* would be associated with a good response to TNFi. Kayakabe et al. measured by ELISA TNF-*α*, IL-1*β*, and IL-6 in supernatant of LPS-stimulated whole blood cultures in 41 RA patients before anti-TNF therapy [48]. This method evaluated monocytic cytokine production. IL-1*β* production at baseline was higher in 6-month responders (median IQR 10.0 (5.1–93.1) versus 3.5 (1.5–9.4) pg/mL for nonresponders). Another study using an in vitro bioassay, based on the induction of IL-6 and osteoprotegerin production by synoviocytes in response to TNF-*α*, suggested that good responsiveness to TNFi was associated with significantly higher TNF-*α* bioactivity at baseline compared to nonresponding patients [5]. Moreover, TNF-*α* expression in the intimal lining layer and synovial sublining of 143 RA patients were significantly higher in responders than in nonresponders (*P* = 0.047 and *P* = 0.008, resp.) [49]. Those 3 studies support the idea that IL-1 and TNF-*α* activity influences response to TNFi, but these analyses cannot be performed in daily practice for technical reasons. Conversely, simple measures of circulating TNF could not predict response [6, 50, 51].

Higher TNF-*α* production in an individual may also result in poorer response, simply due to a subtherapeutic drug dose. In RISING study, the baseline TNF*α* levels, measured by ELISA, predicted the necessity for dose escalation of IFX therapy in 327 patients with RA [52]. Patients with high baseline TNF had higher DAS28, higher levels of RF, and anti-CCP. Baseline TNF levels greatly affected serum IFX concentrations. In TNF-high patients, the median through serum levels of IFX was below limit of detection at 3 and 6 mg/kg and greater than 2 *μ*g/mL at 10 mg/kg, whereas the levels were approximately 1 *μ*g/mL for each dosage in TNF-low patients. Patients with high TNF had a better clinical response to 10 mg/kg than to 3 or 6 mg/kg. This difference was no significant difference in patients with low-TNF. Thus, TNF*α* at baseline could predict the levels of TNF inhibition required.

In 48 RA patients treated with TNFi, IL-17 assessed by ELISA and Th17 at baseline was significantly higher in 6-month EULAR nonresponders than in responders and was found to increase with TNFi in nonresponders, whereas it decreased in responders [6]. Multiple logistic regression analysis showed that high baseline IL-17 level ≥ 40 pg/mL could significantly predict poor response to TNFi (*P* < 0.01; sensitivity 67% and specificity 83%). Inadequate response to TNFi may reflect TNF-independent but Th17-dominant inflammatory process. This is a very interesting finding, but it has not been validated in another cohort yet.

With a multistep proteomics approach using arthritis antigen array, a multiplex cytokines assay and conventional ELISA, Hueber et al. identified a 24-biomarker signature that enables prediction of positive clinical response to ETN confirmed in three different cohorts (from USA *n* = 29, Sweden *n* = 43, and Japan *n* = 21) with positive predictive values between 58 and 72% and negative predictive values from 63 to 78% (comparison of ≥ACR50 and <ACR20) [53]. These biomarkers included autoantibody profiles (i.e., fibromodulin (246–265), Clusterin (170–188), ApoE (277–296) cit, etc.), and cytokines (including higher levels of GM-CSF, IL-6, IL-1beta, and MCP-1 for responders). Combined autoantibody and cytokine profiles were more predictive for response to ETN in the three cohorts.

### 6.2. Other Biological Therapies

For TCZ, serum (3751 samples), genotype (927 samples), and transcript (217 samples) from five phase 3 trials of TCZ (RADIATE, OPTION, TOWARD, AMBITION, and LITHE) were analyzed to assess their association with treatment response [54]. Higher baseline serum IL-6 levels were significantly associated (*P* < 0.0001) with higher baseline DAS28, erythrocyte sedimentation rate, C-reactive protein, and HAQ. Higher baseline serum IL-6 levels were also significantly associated with better clinical response to TCZ versus placebo in the pooled DMARD inadequate responders (*P* < 0.0001) and in MTX-naive populations (*P* = 0.04). However, the association with treatment response was weak with threefold difference in baseline IL-6 level corresponded to only a 0.17-unit difference in DAS28 at week 16. This was also not true in TNFi inadequate responders, limiting the clinical usefulness of the marker in predicting treatment benefit. IL-6 pathway SNPs and RNA levels were also not strongly associated with treatment response.

Lymphocyte count and BAFF levels were suggested to predict response to RTX [55], but it has not been confirmed [56, 57]. In SMART, low levels of CD27+ memory B cells were significantly associated with good response (OR 1.03 (1.01–1.05), *P* = 0.002) although the association was too small to be used as a predictive biomarker in current practice. A similar trend was found in 103 RA patients with higher levels of memory B cells in nonresponders (OR 0.67 (0.44–1.03), *P* = 0.068) [58]. Conversely, Möller et al. found higher numbers of naive B cells in non- and moderate responders compared to good responders in 35 RA patients [59]. The numbers of memory B cells in the synovial tissue of 24 RA patients at baseline were not predictive of RTX response [60].

CTLA4 is similar to the T-cell costimulatory protein, CD28, and both molecules bind to CD80 and CD86 on antigen-presenting cells. CTLA4 transmits an inhibitory signal to T cells, whereas CD28 transmits a stimulatory signal. CD28 expression on the CD4+ and CD8+ T cells was studied in 32 patients with RA treated with ABA [61]. The overall predictive values of the CD8+CD28− and CD4+CD28− cells for DAS28-CRP remission at 6 months were 0.802 (SE 0.078) and 0.743 (SE 0.089), respectively. Cutoff values were proposed. For prediction of remission at 6 months, a concentration below 87 CD8+CD28− cells/*μ*L had 80.0% sensitivity and 81.8% specificity (Fisher's test: *P* = 0.001), and a concentration below 28 CD4+CD28− cells/*μ*L had a 60.0% sensitivity and 77.3% specificity (*P* = 0.043). Patients having low baseline numbers of CD8+CD28− T cells had at least a 4-fold higher probability of achieving remission within 6 months compared to patients with higher levels of these cells. The same group observed that CD8+CD28− T cells decreased at 48 weeks of treatment with ABA and found that reductions of percentages of circulating CD4+CD28− and CD8+CD28− T cells was directly correlated with the reduction of DAS28-CRP (*r* = 0.58, *P* = 0.014; *r* = 0.47, *P* = 0.059, resp.) [62].

In summary, sera concentrations of TNF*α* or IL-1 cannot be used to predict response to TNFi. Eventually, TNF levels could help to adapt IFX dosage. High IL-17 concentrations could be associated with nonresponse to TNFi, but it needs to be confirmed. Higher IL-6 concentrations are found in TCZ responders, but this measure cannot be used to predict response due to a too low predictive value. Immune cell phenotyping is not a good tool to discriminate responders from nonresponders except maybe for low levels of CD4+ and CD8+ CD28− T cells that could be associated with ABA response if it is confirmed.

## 7. Conclusion

Although many studies have identified predictive factors of response to biological therapies, only a few have been confirmed. Severe diseases (with high HAQ and numbers of previous biological drug failures) are more difficult to treat, whereas high DAS28 at baseline predicts stronger DAS28 decrease regardless the type of treatment. For TNFi, male gender, younger age, nonsmokers, nonobese patients, ACPA or RF negative status predicts a good response to treatment and a few SNPs (PTPRC, EYA4, PDZD2, etc.) are also associated with good clinical outcomes. For TCZ, only IL-6 levels were shown to be associated with response but in a weak manner. For ABA, RF positivity and low levels of CD4+ or CD8+ CD28− could help to predict response. For RTX, RF positivity is strongly associated with the response and can be used in clinical practice to guide clinician choice. Response to RTX also seems to be associated with FCGR3A SNP.

**Figure 1:**
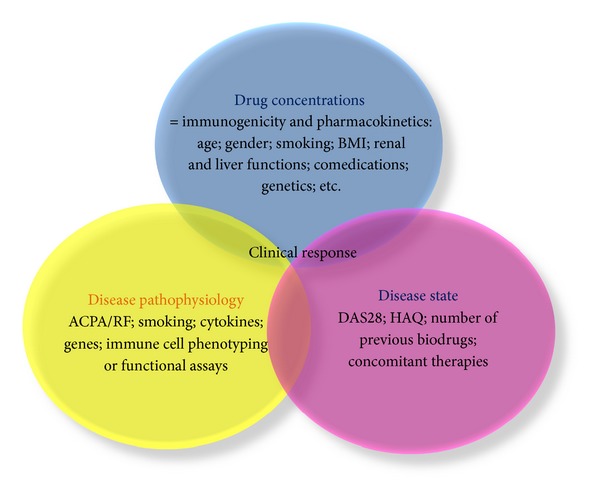
Clinical response depends on many different factors.

Despite some clinical and biological markers (Table 2), prediction of therapeutic response is a goal hard to achieve. The main difficulty is that response depends on at least three different parameters: drug concentrations, disease state, and disease pathophysiology; all of them are depending on many other different factors (Figure 1). Other predictive markers are still required. New approaches are being explored such as epigenetic studies or intracellular signaling response to cytokine stimulation on different immune cells. However, it is likely that no parameter will predict response if taken separately. Future studies should incorporate clinical and biological data to construct discriminating prediction scores that take into account the 3 axes of interindividual variability. Multicenter collaborations will be needed to include a significant number of patients. Finally, it is also important to appreciate the risk of adverse effect occurrence to choose for each patient the drug with the best risk benefit balance.

## Figures and Tables

**Table 1:** Main studies presented in this review.

References	Study, cohort, or first author name	Drugs studied	Study design	Number of RA patients	Endpoint
[6]	Chen	ADA, ETN	Cohort	48	6-month EULAR response
[7]	Kleinert	ADA	Noninterventional study	2,625	12-month DAS28 variation
[8]	ReAct	ADA	Open-label study	6,610	12-week DAS28 remission
[9]	TEMPO	ETN	Randomized controlled double-blind study (MTX or ETN or MTX + ETN)	682	3-year DAS28 remission
[10]	BSRBR	IFX and ETN	Prospective registry	2,879	6-month EULAR response
[11]	GISEA	IFX, ADA, ETN	Retrospective registry	1,565	3-month EULAR response
[12]	REACTION	TCZ	Multicenter retrospective study	229	24-week EULAR response and DAS28 remission
[13]	DANBIO	TCZ	Prospective registry	104	24- and 48-week EULAR response
[14]	ORA	TCZ	Prospective registry	558	6-month EULAR response
[15]	BSRBR	RTX	Prospective registry	540	6-month EULAR response and HAQ improvement
[16]	Klaasen	IFX	Prospective cohort	89	16-week DAS28 variation
[17]	GISEA	IFX, ADA, ETN	Prospective registry	641	12-month DAS28 remission
[19]	Abhishek	IFX, ADA, ETN	Retrospective case control study	395	3-month EULAR response
[20]	Canhão	IFX, ADA, ETN	Prospective cohort	615	EULAR response maintained >3 months in the 1st year
[21]	EIRA	IFX, ADA, ETN	Prospective cohort	535	3-month EULAR response
[29]	Chatzidionysiou	RTX	Observational cohort	2,019	6-month EULAR response
[31]	BSRBR	IFX, ADA, ETN	Prospective registry	642	6-month decrease of DAS28
[32]	Maneiro	TCZ, ABA, RTX	Meta-analysis	23 studies pooled	EULAR and ACR responses
[39]	Jamnitski	ETN	Cohort	89	28-week DAS28 variations
[41]	Cui	IFX, ADA, ETN	Nine RA cohorts	1,283	EULAR response
[42]	Plant	IFX, ADA, ETN	Cohort	1,115	6-month EULAR response
[43]	Plant	IFX, ADA, ETN	Cohort from wellcome trust case control consortium + 2 replication cohorts	566 (+379 and 341)	6-month DAS28 variations
[44]	SMART	RTX	Randomized open trial	111	24-week EULAR response
[45]	Quartuccio	RTX	Cohort	212	4- and 6-month EULAR response
[48]	Kayakabe	IFX, ADA, ETN	Prospective open study	48	24-week EULAR response
[49]	Wijbrandts	IFX	Cohort	149	16-week DAS28 variations
[52]	RISING	IFX	Double-blind randomized trial	327	54-week DAS28 variation and ACR responses
[53]	Hueber	ETN	Three different cohorts	29 + 43 + 21	ACR responses
[54]	RADIATE, OPTION, TOWARD, AMBITION and LITHE	TCZ	Five phase 3 trials	3,143	16-week DAS28 variations
[57]	SMART	RTX	Randomized open label	208	24-week EULAR response
[61]	Scarsi	ABA	Cohort	32	6-month remission

ABA: abatacept; ADA: adalimumab; BSRBR: British society of rheumatology biologics register; DANBIO: nationwide registry of biological therapies in Denmark; DAS28: disease activity score 28 joints; EIRA: epidemiologic investigation of rheumatoid arthritis; ETN: etanercept; EULAR: european league against rheumatism; GISEA: Italian group for the study of early arthritis; HAQ: health assessment quality; IFX: infliximab; ORA: Orencia and Rheumatoid Arthritis; ReAct: research in active rheumatoid arthritis; SMART: a study of retreatment with MabThera (rituximab) in patients with rheumatoid arthritis who have failed on anti-TNF alfa therapy; TEMPO: Trial of Etanercept and Methotrexate with radiographic patient outcomes.

**Table 2:** Main predictive factors of response to biological therapy.

Factors associated with good response to	Tumor necrosis factor inhibitors	Tocilizumab	Abatacept	Rituximab
Patients characteristics	Male (C) [7–9] Younger (C) [7, 8] Nonsmoker (C) [10, 19–21] Nonobese for IFX (C) [16, 17] Use of MTX (C) [7, 8, 10, 11]	Older (NC) [12]	Younger (NC) [13]	Male (NC) [15]
Disease characteristics	Low HAQ (C) [7, 10, 17, 20] High DAS28 (C) [7, 8, 17] ACPA or RF negativity (C) [20, 31] Low number of previous biological therapies (C) [8]	Low HAQ and high DAS28 [13]	High DAS28 [14] RF positivity (C) [32]	Low HAQ and high DAS28 [15, 32] RF positivity +++ (C) [32] Low number of previous biological therapies (C) [29]
Immunogenicity	Antidrug antibodies against ADA or IFX for response to ETN (NC) [39]			
Genetic background	PTPRC = CD45 (rs10919563) (C) [41, 42], 7 SNPs including EYA4 (rs17301249) and PDZD2 (rs1532269) (NC) [43]			158VV FCGR3A in European countries (C) [44, 45]
Cytokines and immune cells	High TNF bioactivity in blood [5] or in synovium [49] (NC), high LPS-stimulated whole blood IL-1b (NC) [48], low IL-17 (NC) [6] 24-biomarker ETN response signature including autoantibodies and cytokines (C) [53]	High serum IL-6 levels (NC) [54]	Low levels of CD4+ and CD8+ CD28− T cells (NC) [61]	Memory B cells (NC) [57, 58]

C: confirmed; NC: not confirmed. To be confirmed, the data had to be validated at least by two independent teams.

## Abbreviations

ACPA: Anticitrullinated peptide antibodies
ACR: American college of rheumatology
ADA: Adalimumab
BAFF: B-cell activating factor
BMI: Body mass index
CRP: C-reactive protein
CTLA4: Cytotoxic T-lymphocyte antigen 4
DAS28: Disease activity score on 28 joints
BSRBR: British Society for Rheumatology Biologics Register
EIRA: Epidemiological Investigation of Rheumatoid Arthritis
ELISA: Enzyme-Linked Immunoabsorbent Assay
EULAR: European league against rheumatism
ETN: Etanercept
EYA4: Eyes absent homolog 4
FCGR3A: Fragment constant region receptor 3A
GM-CSF: Granulocyte macrophage colony-stimulating factor
GWAS: Genome-wide association studies
HAQ: Health Assessment Quality
HLA: Human leukocyte antigen
HR: Hazard ratio
IFX: Infliximab
IL: Interleukin
LPS: lipopolysaccharide
MCP-1: Monocyte chemoattractant protein 1
MTX: Methotrexate
OR: Odds ratio
ORA: Orencia and Rheumatoid Arthritis
PDZD2: PDZ domain containing protein 2
PTPN22: Protein tyrosine phosphatase, nonreceptor type 22
RA: Rheumatoid arthritis
ReAct: Research in Active RA trial
RNA: Ribonucleic acid
RF: Rheumatoid factor
RTX: Rituximab
sDMARD: Synthetic disease modifying antirheumatic drugs
SE: Standard error
SNPs: Single nucleotide polymorphisms
STAT4: Signal transducer and activator of transcription protein 4
TEMPO: Trial of Etanercept and Methotrexate with Radiographic Patient Outcomes
TCZ: Tocilizumab
Th: T helper
TNF: Tumor necrosis factor
TNFAIP3: Tumor necrosis factor, alpha induced protein 3
TNFi: Tumor necrosis factor inhibitor.
